# Fruit Growth Stage Transitions in Two Mango Cultivars Grown in a Mediterranean Environment

**DOI:** 10.3390/plants10071332

**Published:** 2021-06-29

**Authors:** Alessandro Carella, Giuseppe Gianguzzi, Alessio Scalisi, Vittorio Farina, Paolo Inglese, Riccardo Lo Bianco

**Affiliations:** 1 Department of Agricultural, Food and Forest Sciences (SAAF), University of Palermo, 90133 Palermo, Italy; alessandro.carella@unipa.it (A.C.); giuseppe.gianguzzi@unipa.it (G.G.); alessio.scalisi@agriculture.vic.gov.au (A.S.); vittorio.farina@unipa.it (V.F.); paolo.inglese@unipa.it (P.I.); 2 Tatura SmartFarm, Agriculture Victoria, 255 Ferguson Rd, Tatura, VIC 3616, Australia

**Keywords:** fruit development, fruit gauge, VPD, *Mangifera* *indica*, cell division, cell expansion, ripening

## Abstract

Studying mango (*Mangifera* *indica* L.) fruit development represents one of the most important aspects for the precise orchard management under non-native environmental conditions. In this work, precision fruit gauges were used to investigate important eco-physiological aspects of fruit growth in two mango cultivars, Keitt (late ripening) and Tommy Atkins (early-mid ripening). Fruit absolute growth rate (AGR, mm day^−1^), daily diameter fluctuation (ΔD, mm), and a development index given by their ratio (AGR/ΔD) were monitored to identify the prevalent mechanism (cell division, cell expansion, ripening) involved in fruit development in three (‘Tommy Atkins’) or four (‘Keitt’) different periods during growth. In ‘Keitt’, cell division prevailed over cell expansion from 58 to 64 days after full bloom (DAFB), while the opposite occurred from 74 to 85 DAFB. Starting at 100 DAFB, internal changes prevailed over fruit growth, indicating the beginning of the ripening stage. In Tommy Atkins (an early ripening cultivar), no significant differences in AGR/ΔD was found among monitoring periods, indicating that both cell division and expansion coexisted at gradually decreasing rates until fruit harvest. To evaluate the effect of microclimate on fruit growth the relationship between vapor pressure deficit (VPD) and ΔD was also studied. In ‘Keitt’, VPD was the main driving force determining fruit diameter fluctuations. In ‘Tommy Atkins’, the lack of relationship between VPD and ΔD suggest a hydric isolation of the fruit due to the disruption of xylem and stomatal flows starting at 65 DAFB. Further studies are needed to confirm this hypothesis.

## 1. Introduction

Recently, tropical and sub-tropical crops like mango (*Mangifera indica* L.) have been introduced in Sicily. The region is characterized by Mediterranean climate with mild temperatures, long periods of summer drought and relatively wet winters. This environment can be considered a transition zone between the arid climate of North Africa and the temperate-humid climate of Central Europe [1]. The climate is influenced by the interactions between mid-latitudes and tropical phenomena that make it potentially vulnerable to climate change associated to an increase in temperature and a decrease in precipitation, so as to be identified as one of the most important hot-spots of the last decades [2,3]. Climate change has a major impact also on agriculture [4,5,6], especially if we consider that in these areas some growers are moving their business towards the production of newly introduced exotic fruits of tropical origin [7,8].

The possibility to cultivate mango in non-tropical or sub-tropical areas is subjected to the effect of temperature [9]. Mango cannot be cultivated in areas where the average temperature of the coldest month is less than 15 °C [10], while the optimum growing temperature ranges between 24 and 26 °C, reaching 30 to 33 °C for the flowering and fruit development stages [11,12]. These conditions are satisfied in the coastal areas of Sicily [13], where new orchards have been established [9].

Fruit growth is one of the most important parameters to evaluate the adaptation of a species to particular micro-climatic conditions, because the fruit represents the main sink organ of the plant and it can be considered an optimal indicator of its water and nutrient status [14]. The growth curve of mango fruits follows a sigmoidal pattern [15] and can be obtained non-destructively by measuring fruit length, width and thickness at short intervals along its developmental period [16]. The growth pattern is split in three developmental stages: cell division, cell expansion, and ripening [17,18]. Cell division is a very energy demanding process [19], due to the very fast cell division rate in fruit tissues. Carbohydrate intake is therefore crucial in this phase [20]. Carbohydrates translocated into the fruit are mainly imported from actively photosynthesizing leaves through the phloem [21].

Once this first stage is over, fruits start a linear growth, mainly characterized by the expansion of pulp cells due to water uptake led mainly by osmotic gradients. This stage is strongly influenced by the daily fluctuations of temperature and relative humidity, as well as vapor pressure deficit (VPD), which play an important role on fruit transpiration [22]. Specifically, daily VPD fluctuations drive fruit enlargement during the night and shrinkage during the day [18]. This occurs because during the day, transpiration reduces the xylem water potential, and consequently decreases the xylem flow to the fruit causing it to shrink in diameter. During the evening and the night, the water potential is restored and the fruit returns to its volume or increases it [23,24]. It is common, during this period, that in situations of severe water stress, the plant takes water directly from the fruit via the xylem. This phenomenon is called backflow [25], as also documented in apple and kiwi [26,27,28]. In this regard, the variation in fruit diameter over a time interval represents the net contribution of the import of phloem flows, which are always positive, and xylem flows, which, depending on time of day, can be positive or negative [29]. When VPD is high, leaf and fruit transpiration flows will increase, determining some degree of plant dehydration and causing large fruit diameter fluctuations.

The last stage of development is fruit ripening, in which the fruit becomes physiologically and sexually mature enough to be separated from the mother plant [30]. At this stage, internal and external changes in the fruit texture, flavor, and color are observed [31,32].

Fruit gauges are small dendrometers able to monitor continuously and very precisely the variations of fruit diameter, even at very short intervals. They are based on low-cost linear potentiometers connected to a data-logger device [29]. These instruments have already demonstrated good adaptability to different fruit species.

In kiwi, Morandi et al. [33] used these devices to monitor the development of the fruit in its final stage, determining the contributions of xylem and phloem flows to fruit growth. In another study, Morandi et al. [34] used fruit gauges to evaluate the influence of peach fruit transpiration on daily. The same devices were used to evaluate how the level of irrigation affects the daily growth pattern of pear [35], nectarine [36], orange [37], and olive [38,39] fruit.

Considering the increasing interest in mango cultivation in temperate areas, to date, there is insufficient information about the growth and ripening stages of this fruit, as well as about its daily growth dynamics in Mediterranean environments.

The main aim of this trial was to acquire precise indications on the stages of fruit development of two mango cultivars (‘Keitt’ and ‘Tommy Atkins’) with reference to environmental parameters. The use of fruit gauges aimed to improve basic knowledge of the mango fruit physiology, but at the same time, provided useful information for the development of precise crop management resulting in quality and sustainable productions.

## 2. Materials and Methods

### 2.1. Orchard Characteristics and Plant Material

The experiment was carried out in a commercial orchard of the Cupitur Farm located in Caronia (38°03′ N, and 14°33′ E, 5 m a.s.l.) in northeastern Sicily (Italy) from July to October 2019. In that orchard, there are windbreaks made of cypress plants (*Cupressus sempervirens* L.), and nonwoven fabric windbreaks supported by wooden poles of 5 m high.

The study was performed using 15-year-old mango trees (*Mangifera indica* L.), three of the cultivar ‘Tommy Atkins’ (early- to mid-season ripening) and three of the cultivar ‘Keitt’ (late-season ripening) of similar size and crop load of 0.7 and 1.3 fruits cm^−2^ of trunk cross-sectional area, respectively. Both cultivars were grafted onto Gomera-3 mango rootstock. The planting density was 500 trees ha^−1^, with a spacing of 5 × 4 m. Trees were trained to globe-shaped canopies, reaching 2.5–3 m in height. All the trees received the same conventional cultural cares. Trees were drip irrigated with a seasonal irrigation volume of 3300 m^3^ ha^−1^. Fertilization with N was carried out twice, at the beginning of vegetative growth in early spring, and at fruit set. P, K, and microelements were delivered with the irrigation water throughout the season. Two light pruning operations were carried out, one at the end of winter, before the vegetative resting period, and one after fruit harvest.

### 2.2. Environmental Conditions 

The climate is Mediterranean [40], with average annual temperatures of 17–18 °C and average rainfalls of about 691 mm distributed across 77 days [13,41,42]. The experiment location falls into the upper thermos-Mediterranean lower sub-humid bioclimatic belt [13,43]. Data of average temperature and average humidity were acquired by a PCE-HT71 data-logger placed in the field. Data of daily temperature and relative humidity were used to calculate vapor pressure deficit (VPD).

### 2.3. Fruit Measurements and Experimental Design

Starting at 10 days after full bloom, fruit thickness, width, and length were measured in both cultivars using a digital caliper at two-week intervals.

Fruit diameter was monitored continuously, at 15-min intervals, with the fruit gauges described by Morandi et al. [29] connected to a CR-1000 data logger (Campbell Scientific, Inc., Logan, UT, USA). In each cultivar, measurements were carried out using 12 fruit gauges placed in four fruits from different portions of the canopy in each of three trees per cultivar. Fruit gauges were placed in the two cultivars at different times, as their timing of fruit development differs. Measurements started at 51 days after full bloom (DAFB) in the early-mid ripening ‘Tommy Atkins’, and at 58 DAFB in the late ripening ‘Keitt’.

In ‘Tommy Atkins’, diameter changes were monitored in three different periods: I (20–27 July) from 51 to 58 DAFB; II (3–12 August) from 65 to 74 DAFB; III (24 August–7 September) from 86 to 100 DAFB. Measurements of ‘Tommy Atkins’ covered 30 days, corresponding to about 30% of its entire fruiting period.

In ‘Keitt’, diameter changes were monitored in four different periods: I (27 July–2 August) from 58 to 64 DAFB; II (12–23 August) from 74 to 85 DAFB; III (7–21 September) from 100 to 113 DAFB; IV (21 September–3 October) from 114 to 126 DAFB. Measurements of ‘Keitt’ covered 52 days, corresponding to about 35% of its entire fruiting period.

### 2.4. Fruit Development Parameters

Data recorded by the data-logger were processed by graphical analysis. Fruit absolute growth rate (AGR, mm day^−1^) was estimated by calculating the slope of the diameter changes measured by fruit gauges in each monitored period.

The average daily fluctuation of fruit diameter (ΔD, mm) was also calculated for each development stage. It is mainly related to fruit water exchanges (via xylem and transpiration) and more evident during the cell expansion period. Finally, it was calculated a fruit development index, obtained from the ratio between AGR and ΔD. This index was useful to detect the shifts from cell division stage to cell expansion stage to ripening stage. The rationale behind this was that a relatively high index would be associated to cell division, where a low ΔD and a high AGR are expected (growth due to small and actively dividing cells); an intermediate index would be associated to cell expansion, where both high ΔD and AGR are expected (growth due mainly to cell water influx); a relatively low index would be associated to fruit ripening, where a medium to high ΔD and a low AGR are expected (very low growth and some cell water exchanges). To evaluate the influence of the environment on fruit development, VPD and ΔD were also related.

### 2.5. Statistical Analysis

Linear regression analysis was performed on fruit gauge data to estimate AGR in each fruit development stage, using Sigmaplot 12.0 (Systat Software Inc., Chicago, IL, USA) procedures. Sigmaplot regression analysis procedures were also used to test the relationships between ΔD and VPD and evaluate the influence of the environment on fruit growth. Differences of AGR, ΔD, and development index (AGR ΔD^−1^) among measurement periods were tested using one-way analysis of variance (ANOVA). The means were compared by Tukey’s multiple comparison test at the 0.05 significant level using Systat statistical software version 13 (Systat Software Inc.). As a temporal reference, it was used the central day of the interval of each period. For ‘Tommy Atkins’, the reference days were: 56 DAFB (I); 69 DAFB (II); 93 DAFB (III). For ‘Keitt’, the reference days were: 61 DAFB (I); 80 DAFB (II); 107 DAFB (III); 120 DAFB (IV). 

## 3. Results and Discussion

### 3.1. Climate Data

During the experiment, the average temperature was 25.9 °C, with a maximum temperature of 35.9 °C reached on 22 July, and a minimum temperature of 18.0 °C reached on 3 October. The average relative humidity (RH) of the area was 68.1%, with a minimum value of 32.0% recorded on 8 August. The maximum RH was 98.9% recorded on 4 September during the rainfall events (>120 mm) that occurred in the period between the end of August and the beginning of September.

**Figure 1:**
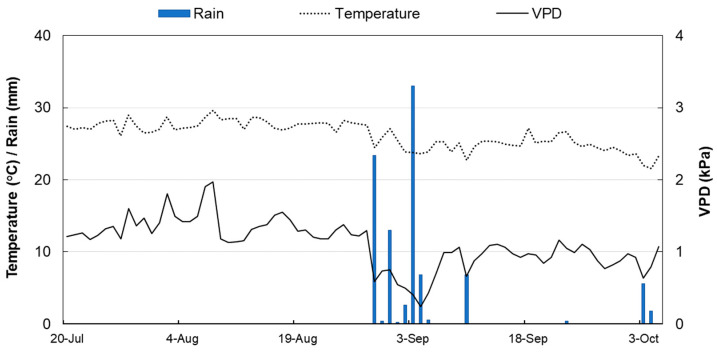
Trends of daily temperature, VPD, and rainfall at Caronia, northern Sicily (38°03′ N, and 14°33′ E, 5 m a.s.l.) during the trial period.

At the beginning of August, the weather was hot and dry, with relatively high VPD values reaching 1.5–2 kPa (on 8 August). VPD shown a constant pattern from 20 July to 28 August and from 7 September to the end of the trial (5 October), with daily minimum values of 0.64 kPa and maximum values of 2 kPa. Between 29 August and 6 September, a heavy rain event caused a rapid drop of daily VPD to 0.21 kPa (Figure 1).

### 3.2. Fruit Growth

**Figure 2:**
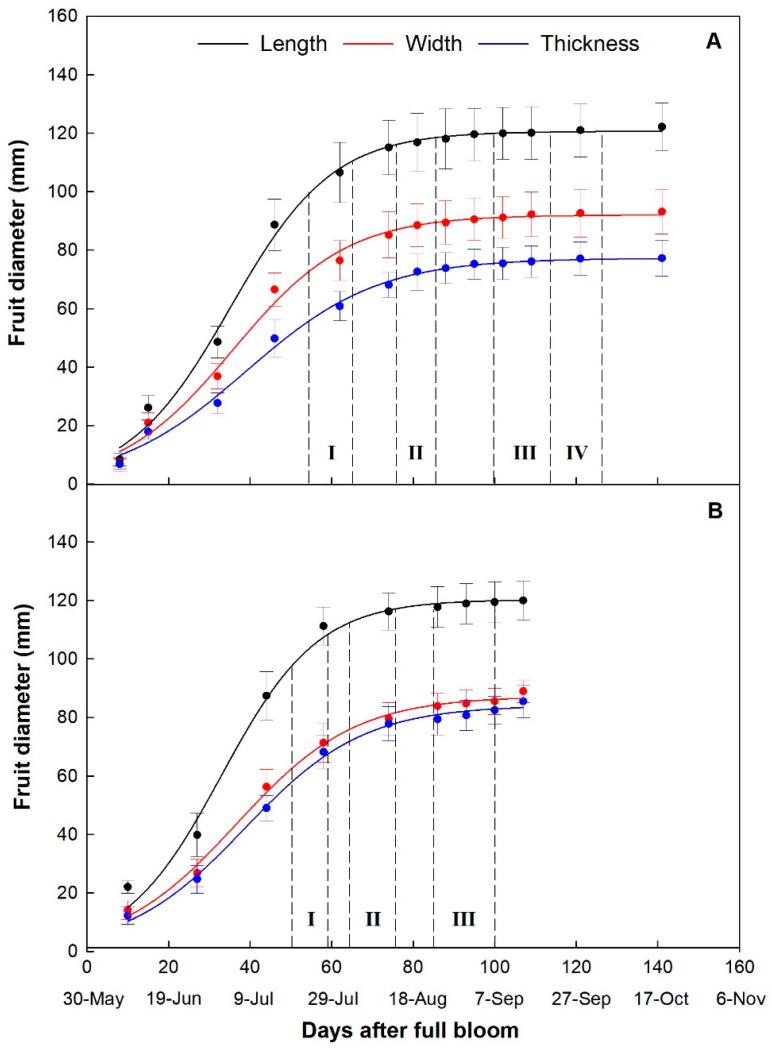
Fruit growth curve of ‘Keitt’ (**A**) in the four observation periods (I, II, III, IV), and ‘Tommy Atkins’ (**B**) in the three observation periods (I, II, III) monitored with a digital caliper. In ‘Keitt’, Length = 120.62/(1 + e^(−(DAFB−35.11)/12.56)^); R^2^ = 0.99. Width = 92.04/(1 + e^(−(DAFB−35.84)/14.01)^); R^2^ = 0.99. Thickness = 77.23/(1 + e^(−(DAFB−39.01)/16.11)^); R^2^ = 0.99. In ‘Tommy Atkins’, Length = 120.31/(1 + e^(−(DAFB−32.95)/11.81)^); R^2^ = 0.99. Width = 87.25/(1 + e^(−(DAFB−36.66)/14.56)^); R^2^ = 0.99. Thickness = 84.13/(1 + e^(−(DAFB−38.66)/14.56)^); R^2^ = 0.99.

The typical sigmoidal fruit growth pattern was observed in fruits of both cultivars (Figure 2). In ‘Keitt’, the growth in length was the most rapid followed by width and finally by thickness, resulting in the characteristic flattened shape of the fruit (Figure 2A) [44]. In ‘Tommy Atkins’, although length was also the most rapidly growing fruit dimension, fruit width and thickness showed very similar trends, resulting in a rounder shape typical of this cultivar (Figure 2B).

The growth curves highlighted the expected difference in the length of the fruiting period in the two cultivars, lasting about 105 days in ‘Tommy Atkins’ and about 140 days in ‘Keitt’. This difference consisted mostly in a longer ripening stage in ‘Keitt’ than in ‘Tommy Atkins’, because, even though both cultivars have similar polygalacturonase activity, ‘Keitt’ retains more total pectin at the beginning of the ripening stage than ‘Tommy Atkins’ [45].

**Figure 3:**
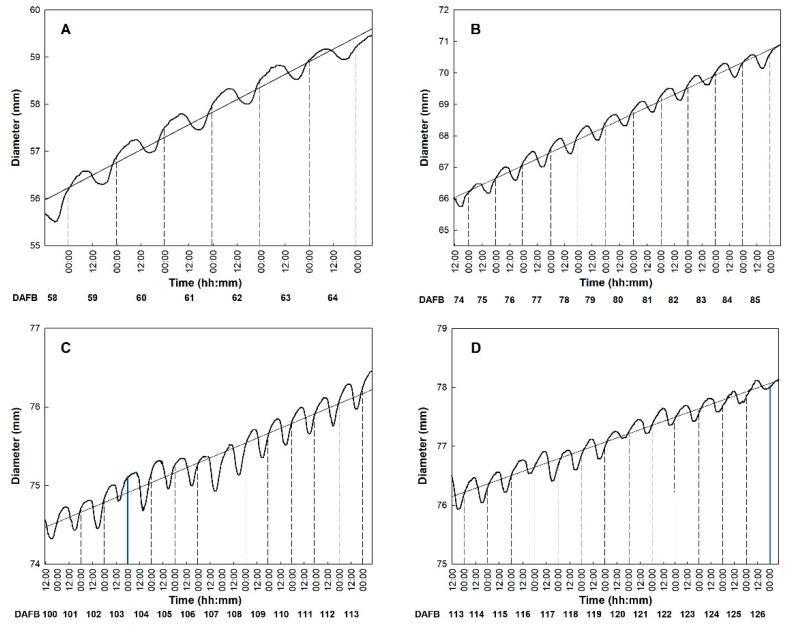
Trends of diametric growth of ‘Keitt’ mangos in the four observation periods monitored by fruit gauges every 15 min from 58 to 64 DAFB (**A**), from 74 to 85 DAFB (**B**), from 100 to 113 days DAFB (**C**), and from 114 to 126 DAFB (**D**). The blue lines indicate days of rainfall. Period I: Diameter = 0.535DAFB − 23,297; R^2^ = 0.957; *p* < 0.001. Period (**II**): Diameter = 0.410DAFB – 17,864; R^2^ = 0.978; *p* < 0.0001. Period (**III**): Diameter = 0.126DAFB − 5434; R^2^ = 0.910; *p* < 0.001. Period (**III**): Diameter = 0.142DAFB − 6137; R^2^ = 0.948; *p* < 0.001.

The use of fruit gauges allowed for the detection of diameter variation and monitoring of growth rate during the 24 h (Figure 3). The fruit gauges recorded diameter fluctuations throughout the day, which continued with a similar fashion in all the monitoring periods. In fact, from about 18:00 to about 8:00 of the next day, there was a rapid increase of fruit diameter followed by a sharp decrease during the hottest hours, with a different net diameter increase (growth rate) depending on the period of observation. This is in line with what is explained by the model of Léchaudel et al. [46] and Faust [47], which emphasizes a negative correlation between transpiration and water potential in mango fruit. In detail, the transpiration rate is maximum during the hottest hours, when xylem water potential reaches its minimum, while during the evening and the night water potential is restored, and fruits gradually expand to reach sizes larger than the initial.

**Figure 4:**
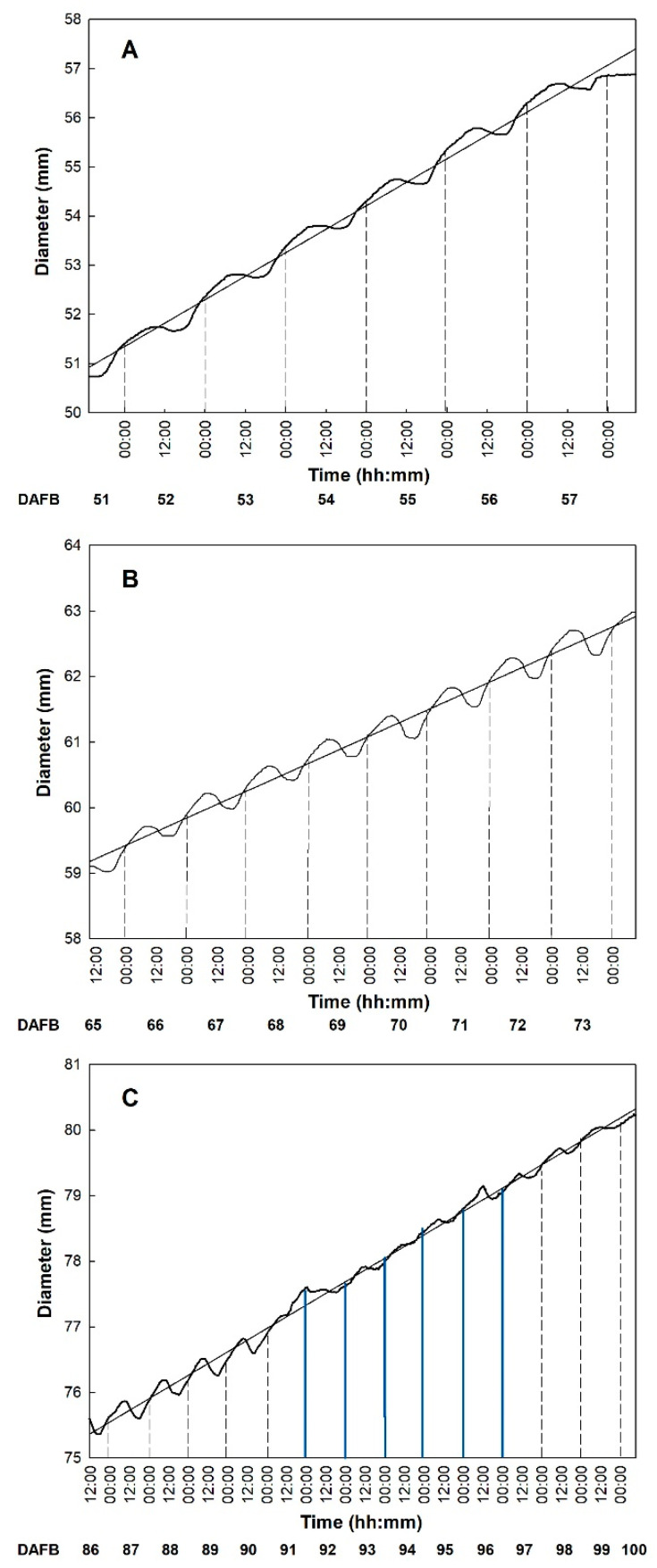
Trends of diametric growth of ‘Tommy Atkins’ mangos in the three observation periods monitored by fruit gauges every 15 min from 51 to 58 DAFB (**A**), from 65 to 74 DAFB (**B**), and from 86 to 100 DAFB (**C**). The blue lines indicate days of rainfall. Period I: Diameter = 1.010DAFB − 50,888; R^2^ = 0.991; *p* < 0.001. Period II: Diameter = 0.417DAFB − 18,141; R^2^ = 0.989; *p* < 0.001. Period (**III**): Diameter = 0.357DAFB – 15,513; R^2^ = 0.995; *p* < 0.001.

In ‘Keitt’, fruit diameter started shrinking at 8:30–9:30 and continued until 17:30–18:00. During this time of the day, fruits may lose water, and consequently volume in two ways: directly, because of the fruit transpiration, and indirectly, via xylem (backflow). This occurs when the leaf transpiration is rather high, especially during warm days and high VPD, and the water supply from the roots is insufficient to counteract the water shortage. In these cases, water might flow outward from the fruit according to the water potential gradient [25]. Thus, fruit shrinkage is caused by a negative water balance because water losses cannot be compensated by phloem inputs. From 18:00 onward fruit diameter increased until the morning, with the fruit generally reaching a larger size than the previous day (Figure 3). This size difference between consecutive days represents the net daily growth and can be mainly associated to an accumulation of dry matter, including a pool of organic and inorganic molecules that become part of the cellular structures of the fruit (Figure 4). Only in period III, between 91 and 96 DAFB (Figure 4 III), reduced and irregular diameter fluctuations were detected. This occurred in correspondence with intense rainfall events between 29 August and 3 September, i.e., when RH was >90% and VPD reached 0.21 kPa (Figure 1). According to Torres Ruiz et al. [48] supplying water when kiwi vines are experiencing stressing environmental conditions, e.g., very high transpiration rates, can influence fruit volume growth in the following days, causing a marked reduction of daily fluctuations. About two days later, RH, temperature and VPD values returned to the typical values of the period and fruits gradually resumed regular daily fluctuations and growth (Figure 4 III).

Similarly, in ‘Tommy Atkins’, a diametric decrease was observed during the warmest part of the day, starting between 8:30 and 9:15 and ending between 17:00 and 17:45. Subsequently, a constant increase in diameter was recorded during the observation periods (Figure 4). Only in period III, between 91 and 96 DAFB (Figure 4 III), reduced and irregular diameter fluctuations were detected. This occurred in correspondence with intense rainfall events between 29 August and 3 September, i.e., when RH was >90% and VPD reached 0.21 kPa (Figure 1). According to Torres Ruiz et al. [48], supplying water when kiwi vines are experiencing stressing environmental conditions, e.g., very high transpiration rates, can influence fruit volume growth in the following days, causing a marked reduction of daily fluctuations. About two days later, RH, temperature and VPD values returned to the typical values of the period and fruits gradually resumed regular daily fluctuations and growth (Figure 4 III).

### 3.3. Fruit Development

**Figure 5:**
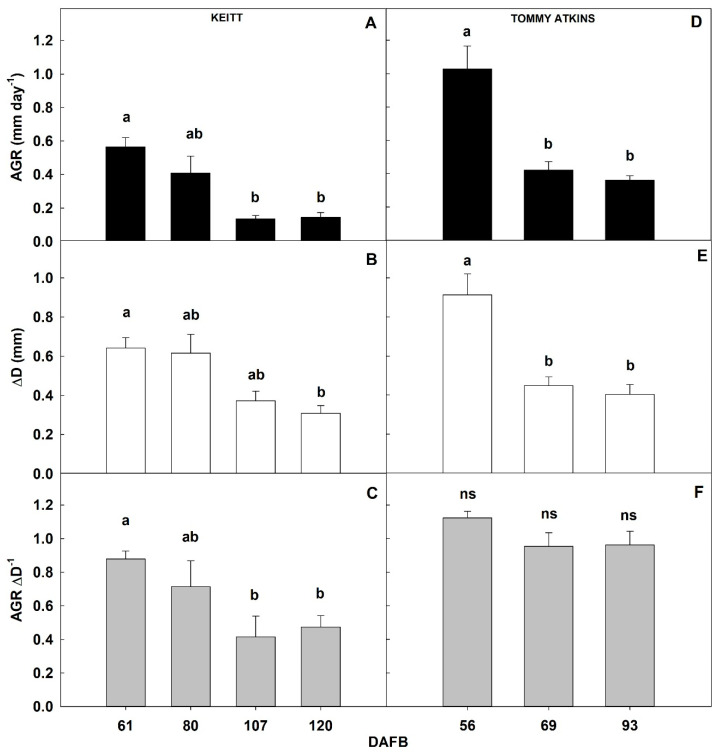
Fruit absolute growth rate (AGR), daily diameter fluctuations (ΔD), and development index (AGR ΔD^−1^) of ‘Keitt’ (**A**–**C**) and ‘Tommy Atkins’ (**D**–**F**) mango during the monitoring periods (Keitt: 61, 80, 107, 120 DAFB. Tommy Atkins: 56, 69, 93 DAFB). DAFB = days after full bloom.

In both cultivars, fruit AGR decreased significantly with progressing development (Figure 5). Specifically, in ‘Keitt’, AGR was highest in period I, with values reaching 0.53 ± 0.05 mm day^−1^. A significant and similar reduction was observed in periods III and IV, while AGR in period II was intermediate (Figure 5A). This suggests a substantial decrease in dry matter accumulation rate by the fruit during its development. It also indicates that during period I, fruits were still in an active growth phase, probably due to cell expansion mechanisms, strictly dependent on carbohydrate accumulation and water recall by osmosis. Subsequently, the reduction in growth confirmed that period II was a transition period between the end of cell expansion and the beginning of ripening. A similar decrease of AGR during mango fruit development was shown by Lechaudel in ‘Lirfa’ [49] and by Dambreville in ‘Cogshall’ and ‘José’ [50]. Following periods occurred at the time of full drupe maturation (Figure 5A).

In ‘Tommy Atkins’, AGR was highest also in period I with an average of 1.01 ± 0.14 mm day^−1^, showing a high net daily growth, also visible in the first period of the sigmoidal curve between 20 and 60 DAFB (Figure 2B). In subsequent periods, there was a significant and similar reduction in fruit growth of about 60%, with values of 0.42 ± 0.05 mm day^−1^ and 0.36 ± 0.02 mm day^−1^ in period II and III, respectively (Figure 5D). In this case, fruits monitored in period I were actively growing, most likely by cell expansion, while fruits at periods II and III were already at the ripening stage.

In ‘Keitt’, also ΔD showed a significant reduction over the four periods of fruit development, going from 0.64 ± 0.05 mm in period I, to 0.30 ± 0.04 mm in period IV (Figure 5B). This indicates that fruits monitored in periods III and IV were not at cell expansion stage as cell expansion depends mainly on water inflow that would have caused marked daily diameter fluctuations (Figure 5B). Decreases in ΔD could be associated with lower water exchanges from the fruit to either the atmosphere or the rest of the plant at more advanced developmental stages. These could be determined by the beginning of the ripening stage, in which the water exchanges between the fruit and the environment (transpiration) are gradually reduced, probably due to the thickening of the peel cuticle [51,52,53].

Also in ‘Tommy Atkins’, marked diameter fluctuations were detected in period I (0.91 ± 0.11 mm). However, these were more than halved in periods II and III dropping to 0.45 ± 0.04 and 0.40 ± 0.05 mm, respectively (Figure 5E). This is a second piece of evidence indicating that the fruit in period I was still in an active growth phase due to cell expansion mechanisms associated to water exchanges. The reduction of ΔD in periods II and III could be due to a reduction in fruit transpiration, possibly driven by cuticular thickening phenomena during ripening [54], or to a lower xylem communication with the plant. Indeed, cuticular conductance values in mango can vary depending on the microclimatic conditions of fruit growth [24], thus limiting transpiration phenomena [55]. In this regard, Léchaudel et al. [56] showed that the rate of water accumulation in mangoes decreases when the dry matter of the fruit increases. All these phenomena (reduction of growth, reduction of the transpiration rate by cuticular thickening, xylem isolation) may be associated with the beginning of the ripening phase, which is also characterized by the activation of metabolic activities determining physiological, biochemical, and organoleptic changes [32,57].

**Figure 6:**
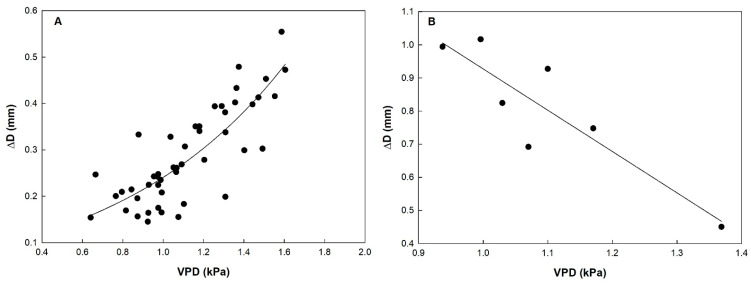
Relationship between vapor pressure deficit (VPD) and daily fluctuations in fruit diameter (ΔD) in ‘Keitt’ over the four monitoring periods (**A**), and ‘Tommy Atkins’ (**B**) mango at monitoring period I. Keitt: ΔD = 0.076 × e^(1.1579VPD)^; R^2^ = 0.679; *p* < 0.001. Tommy Atkins: ΔD = 2.175 − 1.248VPD; R^2^ = 0.788; *p* = 0.008.

In order to evaluate the effect of microclimate conditions on fruit growth dynamics, ΔD was related to VPD. In ‘Keitt’, a direct exponential relationship was found between the two parameters over the four monitoring periods, i.e., as VPD increased, the amplitude of daily fluctuations in fruit diameter rapidly increased (Figure 6A). In this case, VPD seems to be the main driving force determining fruit diameter fluctuations. Morandi et al. [23] found a similar behavior in peach fruit showing a direct relationship between transpiration rate (directly linked with diameter fluctuations) and VPD at cell division and cell expansion stages, demonstrating a tight coupling of fruit transpiration to environmental conditions during these developmental stages.

In ‘Tommy Atkins’, on the other hand, a linear inverse relationship between ΔD and VPD was found only in period I (Figure 6B). Fruits of the Tommy Atkins cultivar have a relatively low rate of transpiration compared with other mango cultivars, due to a cuticle structure characterized by a relatively high wax content, a limited number of lenticels [51] and a high density of resin ducts [58]. This along with a possible leaf stomatal closure in response to high VPD may explain the decrease of daily diametrical oscillations as VPD increased. Already at period II, however, no relationship (*p* = 0.083) between the two parameters was found. This could support the hypothesis that in this period, the fruit was entering the ripening phase and was becoming isolated at the hydric level, i.e., interrupting xylem and stomatal flows, and non-dependent on VPD variations. This phenomenon was most evident in period III, where the absence of the relationship between ΔD and VPD was confirmed (*p* = 0.278), supporting the hypothesis that during ripening, fruits limit water exchanges with the atmosphere (no transpiration) and with the rest of the plant by xylem backflow. In fact, data recorded in period III showed no significant changes in diameter. In kiwi, this has been attributed to a drop in the daily transpiration rate, as fruits stop xylem inflow in the last phase limiting the formation of pressure gradients ideal for water movement [33]. According to Nordey [52], in ‘Cogshal’ mango, changes in xylem flow are related to a decrease in water conductivity of xylem vessels, driven by vessel occlusion. Also in fruits of other species, including kiwi [27,54], apple [26], cherry [59] and grapes [60], an occlusion of xylem vessels was observed as the conduction of these tissues during particular climatic conditions could imply large water losses by backflow [59]. As mentioned above, ‘Tommy Atkins’ fruits have very low cuticular conductance compared to other varieties, especially at the ripening stage, and this may explain the different behavior of ‘Tommy Atkins’ and ‘Keitt’ in response to VPD [61]. In particular, ‘Keitt’ presents greater proportion of amorphous zone than ‘Tommy Atkins’, due to a faster cuticular degradation, increasing fruit skin water permeability.

The ratio between AGR and ΔD, or development index, was calculated to understand what the prevailing mechanism between cell division and expansion in the growth model was, assuming that a large ΔD would be mainly due to intense water exchanges typical of the cell expansion mechanism. Specifically, in ‘Keitt’, the development index was significantly higher in period I than in periods III and IV, with intermediate values in period II (Figure 5C). Considering that both parameters followed similar decreasing trends and that AGR values were very low in periods III and IV, it can be stated that in period I, cell division may have prevailed over cell expansion, while in period III and IV internal changes prevailed over fruit growth, typical of the fruit ripening stage. Period II fell into an intermediate stage where growth gradually slowed down and cell expansion may have prevailed over cell division. The transition stage between periods I and III (65–99 DAFB) can be considered relatively long in this mid- to late-ripening cultivar (Figure 5C). In ‘Tommy Atkins’, AGR and ΔD showed the same trends resulting in no significant differences in development index among monitoring periods (Figure 5F). Since some growth was still present until fruit harvest (although at a slower rate than in period I), we can assume that both cell division and expansion coexisted at constant rates during all three monitoring periods. This indicates on one side, that this early-ripening cultivar keeps growing until the end of fruit development, and on the other side, that a period of greater cell division than cell expansion mechanism must occur before 56 DAFB, if ever.

## 4. Conclusions

This paper describes unpublished information about the growth of mango fruit in a Mediterranean environment and may represent a solid ground for further investigations. The use of fruit gauges on drupes allowed the monitoring of diametric variations over time in response to environmental and physiological conditions of two important cultivars such as Keitt and Tommy Atkins. Thanks to the identification of the different growth stages, and more importantly of the moments when either cell division or cell expansion prevails during fruit development, the optimal time for the application of specific management practices (fertilization, irrigation, fruit thinning) could be established.

The precise identification of the beginning of the ripening phase is also very useful to maximize fruit quality, for example by applying deficit irrigation with nearly no risk to compromise final fruit size.

The association between environmental conditions and fruit growth also indicated that ‘Keitt’ fruit growth takes advantage of increasing VPD, suggesting a good adaptation of this cultivar to Mediterranean environments. The same does not seem to be true for ‘Tommy Atkins’, acting as a genotype sensitive to quick changes in VPD and most likely to the dry conditions of Mediterranean summers. Further scientific work on this track will have to confirm or disprove the fruit hydric isolation hypothesis formulated in this study during the later fruit development stages.

## Data Availability

Data available on request.
